# Restricting Temptations: Neural Mechanisms of Precommitment

**DOI:** 10.1016/j.neuron.2013.05.028

**Published:** 2013-07-24

**Authors:** Molly J. Crockett, Barbara R. Braams, Luke Clark, Philippe N. Tobler, Trevor W. Robbins, Tobias Kalenscher

**Affiliations:** 1 Behavioural and Clinical Neuroscience Institute and Department of Psychology, University of Cambridge, Cambridge CB1 3EB, UK; 2 Laboratory for Social and Neural Systems Research, Department of Economics, University of Zürich, 8006 Zürich, Switzerland; 3 Wellcome Trust Centre for Neuroimaging, University College London, London WC1N 3BG, UK; 4 Institute of Psychology, Leiden University, 2311 EZ Leiden, the Netherlands; 5 Comparative Psychology, Institute of Experimental Psychology, Heinrich Heine University Düsseldorf, 40225 Düsseldorf, Germany; 6 Cognitive and Systems Neuroscience, Swammerdam Institute for Life Sciences, University of Amsterdam, 1098 XH Amsterdam, the Netherlands

## Abstract

Humans can resist temptations by exerting willpower, the effortful inhibition of impulses. But willpower can be disrupted by emotions and depleted over time. Luckily, humans can deploy alternative self-control strategies like precommitment, the voluntary restriction of access to temptations. Here, we examined the neural mechanisms of willpower and precommitment using fMRI. Behaviorally, precommitment facilitated choices for large delayed rewards, relative to willpower, especially in more impulsive individuals. While willpower was associated with activation in dorsolateral prefrontal cortex (DLPFC), posterior parietal cortex (PPC), and inferior frontal gyrus, precommitment engaged lateral frontopolar cortex (LFPC). During precommitment, LFPC showed increased functional connectivity with DLPFC and PPC, especially in more impulsive individuals, and the relationship between impulsivity and LFPC connectivity was mediated by value-related activation in ventromedial PFC. Our findings support a hierarchical model of self-control in which LFPC orchestrates precommitment by controlling action plans in more caudal prefrontal regions as a function of expected value.

## Highlights

• Precommitment is the voluntary restriction of access to temptations
• Precommitment is a more effective self-control strategy than willpower
• Precommitment engages LFPC; willpower engages DLPFC and PPC
• During precommitment, LFPC increases connectivity with DLPFC and PPC

## Teaser

Crocket et al. show that during precommitment, which involves voluntarily restricting access to temptations, lateral frontopolar cortex is activated and increases functional connectivity with dorsolateral prefrontal and posterior parietal cortex—the same regions associated with actively resisting temptations.

## Introduction

Preventing temptations from derailing long-term goals is one of the most universal and challenging problems faced by humans. Because the subjective value of a reward declines as the delay to its receipt increases (a process known as “temporal discounting”; Kable and Glimcher, 2007; Kalenscher and Pennartz, 2008), people are often lured toward choosing small immediate rewards over larger delayed ones, even when such choices are clearly against one’s best interest. Overcoming the temptation to choose immediate (but inferior) rewards requires self-control (Ainslie, 1974; Hare et al., 2009). Struggles with self-control pervade daily life and characterize an array of dysfunctional behaviors, including addiction, overeating, overspending, and procrastination.

Self-control can be implemented in various ways. The bulk of research on self-control has focused on the effortful inhibition of impulses, or willpower (also known as “delay of gratification”; Mischel et al., 1989; Metcalfe and Mischel, 1999; Muraven and Baumeister, 2000). People are often able to successfully resist temptations even from a very young age (Mischel et al., 1989); however, willpower is far from bulletproof. Research has shown that willpower is less successful during “hot” emotional states (Metcalfe and Mischel, 1999; Loewenstein and O’Donoghue, 2004) and may be vulnerable to depletion over time (Muraven and Baumeister, 2000).

But willpower is not the only means by which people resist temptations. One notable alternative self-control strategy is precommitment, in which people anticipate self-control failures and prospectively restrict their access to temptations (Rachlin and Green, 1972; Ainslie, 1974; Wertenbroch, 1998; Ariely and Wertenbroch, 2002; Kalenscher and Pennartz, 2008; Fujita, 2011; Elster, 2000). Examples of precommitment include avoiding purchases of unhealthy food items and locking money away in savings accounts with hefty early withdrawal fees. Notably, precommitment often involves imposing costs for deviating from long-term goals. Wertenbroch (1998) demonstrated that people ration their access to “vices” like cigarettes and junk foods by purchasing them in smaller quantities, even though they could save money by purchasing them in bulk. Another study showed that students self-imposed costly deadlines to avoid procrastination (Ariely and Wertenbroch, 2002). That people do this suggests they are sometimes aware of potential temptations, which makes (costly) precommitment decisions more valuable in the long run relative to unconstrained decisions, which are vulnerable to (more costly) self-control failures.

Even though precommitment is widely used as a self-control strategy outside of the laboratory, and has been the subject of extensive theoretical consideration (Elster, 2000), compared to willpower it has received far less attention from the empirical behavioral sciences (Fujita, 2011), and the neural mechanisms of precommitment remain unknown. In the current study, we developed a behavioral method to directly test the effectiveness of precommitment relative to willpower. We used this measure in conjunction with fMRI to investigate the neural mechanisms of precommitment and its relationship to other varieties of self-control.

Previous studies of the neural basis of self-control have focused primarily on willpower. These studies have consistently implicated the dorsolateral prefrontal cortex (DLPFC), inferior frontal gyrus (IFG), and posterior parietal cortex (PPC) in the effortful inhibition of impulses during self-controlled decision making (McClure et al., 2004, 2007; Hare et al., 2009; Figner et al., 2010; Kober et al., 2010; Essex et al., 2012; Luo et al., 2012). These findings converge with those of studies employing measures of the ability to inhibit prepotent motor responses, which also implicate the DLPFC and IFG (Aron et al., 2004; Chikazoe et al., 2007; Simmonds et al., 2008; Cohen et al., 2012). In line with these studies, we expected to find increased activation in DLPFC, IFG, and PPC when subjects deployed willpower to actively resist temptations.

Meanwhile, the neural basis of self-control by precommitment remains unexplored. Precommitment is nonnormative, in the sense that a rational decision maker with time-consistent preferences should never restrict his choice set. But precommitment is adaptive when willpower failures are expected. Thus, an optimal precommitment strategy should require information about the likelihood of willpower failures. One computationally plausible neural mechanism is a hierarchical model of self-control in which an anatomically distinct network monitors the integrity of willpower processes and implements precommitment decisions by controlling activity in those same regions. The lateral frontopolar cortex (LFPC) is a strong candidate for serving this role. A recently proposed framework of executive decision making places frontopolar cortex at the top of a cognitive control hierarchy, enabling goal pursuit by orchestrating diverging action plans represented in caudal and lateral prefrontal regions (Burgess et al., 2007; Koechlin and Hyafil, 2007; Tsujimoto et al., 2011). Activity in LFPC is associated with prospective valuation and counterfactual thinking, processes that are critical for comparing alternative courses of action (Daw et al., 2006; Burgess et al., 2007; Koechlin and Hyafil, 2007; Boorman et al., 2009, 2011; Rushworth et al., 2011; Tsujimoto et al., 2011). At the same time, LFPC is implicated in metacognitive appraisal and the assessment of confidence in both perceptual and value-based decisions (De Martino et al., 2013; Fleming et al., 2010) and has recently been suggested to represent anticipatory utility during intertemporal choice (Jimura et al., 2013). Based on these studies, we hypothesized that LFPC would be activated during decisions to precommit and would show increased functional connectivity with regions involved in willpower.

**Figure 1:**
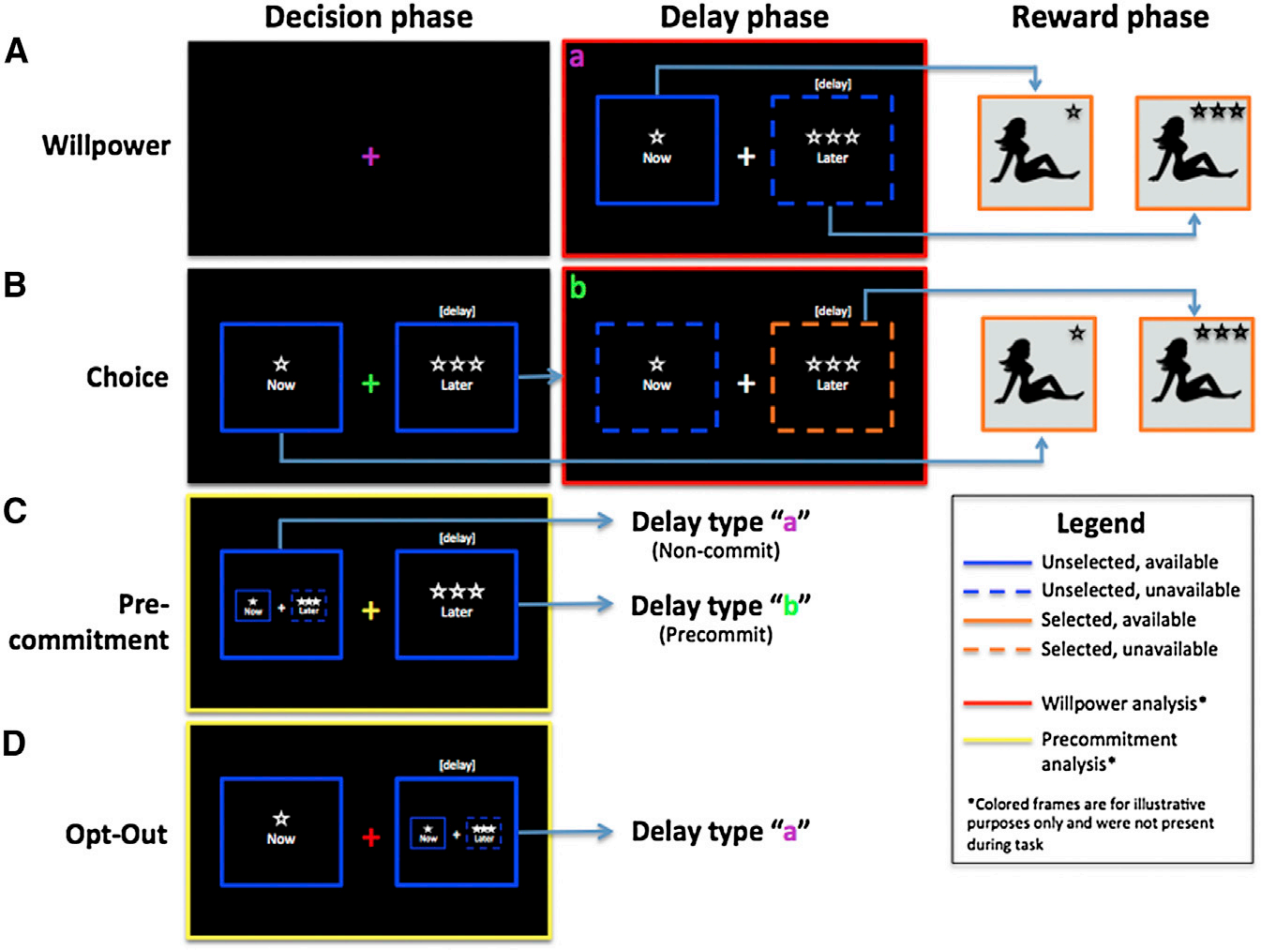
Experimental Task Conditions Participants completed all four conditions, presented in random order. Each task consisted of a decision phase (4,000 ms), a delay phase (0–10,000 ms), and a reward phase (2,500 ms). Participants chose between smaller-sooner (SS) and larger-later (LL) visual rewards. Delay length was indicated above the LL option. Solid lines indicate reward available for selection, while dashed lines indicate reward that is unavailable for selection. Choice options were initially blue and turned orange upon selection. (A) In the Willpower task, participants had to actively resist choosing the available SS reward during the delay phase. (B) In the Choice task, participants simply chose the SS or LL reward during the decision phase. If LL was chosen, the SS was unavailable during the delay phase. (C) In the Precommitment task, participants decided whether or not to make a binding choice for the LL (“commit”). Commitment decisions led to a delay phase identical to that of the Choice task (in which the SS was unavailable), while noncommitment decisions led to a delay phase identical to that of the Willpower task (in which the SS was available). (D) In the Opt-Out task, participants initially decided whether to choose SS or wait for LL. LL decisions led to a delay phase identical to that of the Willpower task, in which the SS was available.

In our study, male participants rated a set of erotic images, and based on their ratings, we constructed personalized stimulus sets consisting of small rewards (images rated slightly above neutral) and large rewards (highly rated images; Table S1 available online). Participants then made choices between viewing a small reward immediately (smaller-sooner reward, or SS) or a large reward after a variable delay (larger-later reward, or LL). We varied the decision characteristics across four experimental task conditions (see Figure 1). In the Willpower task, participants were required to actively resist choosing the SS, which was available throughout the delay period as they waited for the LL. In the Choice task, participants made an initial choice between SS and LL; if they chose LL, they passively waited for the LL during a delay period in which the SS was not available. In the Precommitment task, participants decided whether to remove their ability to choose the SS, thus committing to the LL. In the Opt-Out task, participants decided whether to make a nonbinding choice to wait for the LL; during the delay period, the SS was still available, so they could reverse their choice at any time. All tasks were economically equivalent in terms of rewards, delays, motor responses, and trial durations, and participants were informed of the duration of the delay at the time of choice. Because all trials were equally long, to maximize reward in this paradigm, participants should always choose LL. We examined self-control (here defined as the proportion of LL choices) across our experimental conditions in a behavioral study (Study 1) and an fMRI study (Study 2).

## Results

### Behavioral Task Validation

**Figure 2:**
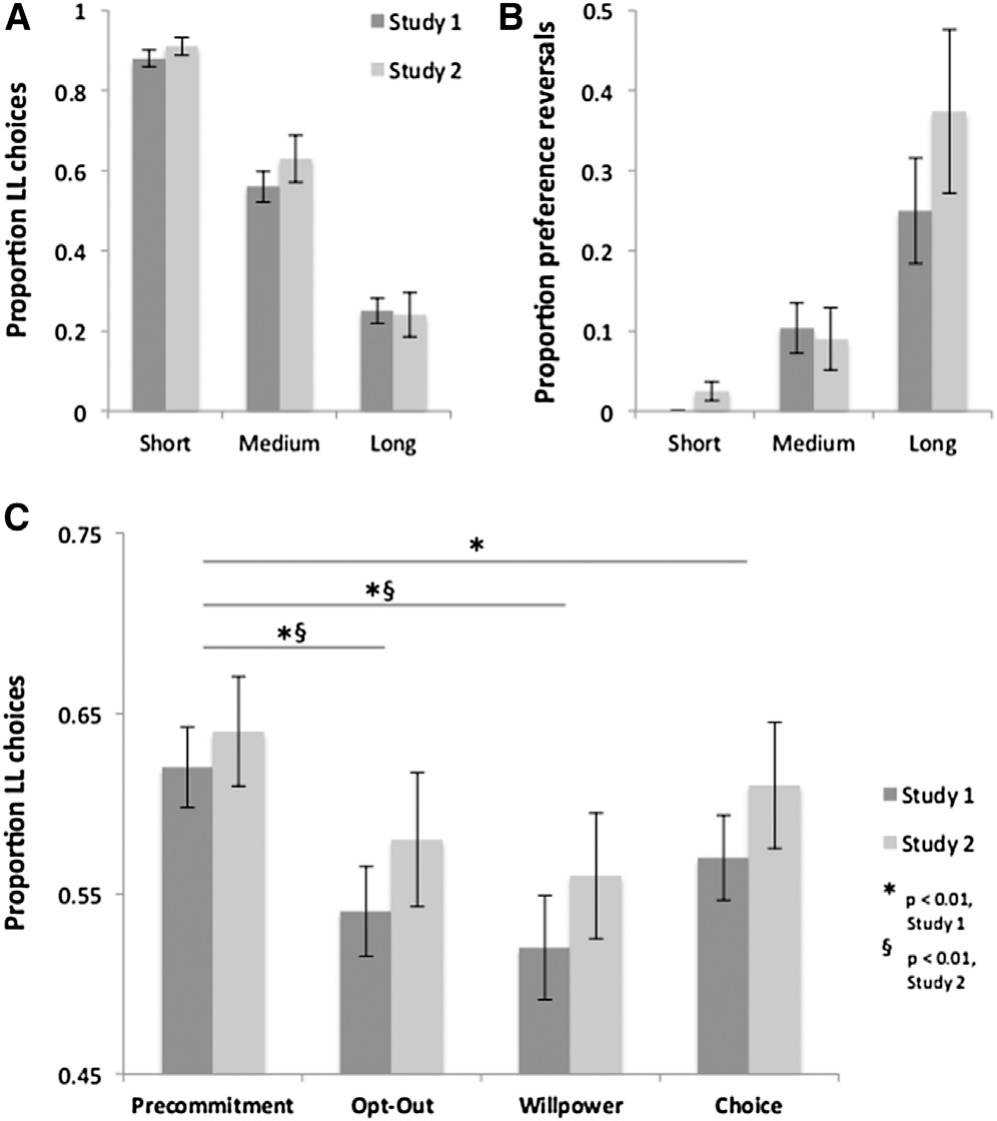
Behavioral Results (A) Self-control (defined as proportion of LL choices) declined with increasing delays. (B) Preference reversals (initial choices for LL, followed by opt-out choices for SS) increased as a function of delay. (C) Self-control differed across task conditions; precommitment facilitated choices for LL in two independent studies. Data are represented as mean ± SEM.

As a manipulation check, we first tested whether self-control decreased as a function of delay. As expected, across all task conditions, participants were more likely to choose LL at short delays, relative to medium delays and long delays (Study 1: F_(2,114)_ = 153.24, p < 0.001; Study 2: F_(2,40)_ = 41.02, p < 0.001; Figure 2A).

To further validate our task as a measure of self-control, we looked for evidence of preference reversals, i.e., instances in which participants initially indicated a preference for LL but later chose SS. Specifically, we examined choices in the Opt-Out task, in which participants could make a nonbinding choice for LL but could choose SS at any point during the delay period. Since the SS was also available during the initial choice (Figure 1D), and at the time of choice participants knew the delay length, choices for SS during the delay period are suboptimal in terms of maximizing reward across time. Figure 2B displays the proportion of SS choices during the delay period conditional on initial choices for LL. We observed a substantial number of preference reversals (one-sample t test, Study 1: t_(57)_ = 4.99, p < 0.0001; Study 2: t_(19)_ = 3.94, p = 0.001), which increased as a function of delay (Study 1: F_(2,82)_ = 12.50, p < 0.0001; Study 2: F_(2,32)_ = 9.64, p = 0.001; Figure 2B). Preference reversals were positively correlated with the proportion of SS choices in the willpower task at a trend level in Study 1 and significantly so in Study 2 (Study 1: r = 0.251, p = 0.068; Study 2: r = 0.648, p = 0.002).

### Precommitment Is a More Effective Self-Control Strategy than Willpower

Despite the fact that all tasks had equivalent rewards and delays, self-control differed across tasks (Study 1: F_(3,171)_ = 17.51, p < 0.001; Study 2: F_(3,60)_ = 7.209, p < 0.001; Figure 2C). The opportunity to precommit improved self-control: participants were more likely to choose LL in the Precommitment task than in the Opt-Out task (Study 1: t_(57)_ = 5.64, p < 0.001; Study 2: t_(19)_ = 3.45, p = 0.003) and the Willpower task (Study 1: t_(57)_ = 5.26, p < 0.001; Study 2: t_(19)_ = 3.58, p = 0.002), as well as the Choice task in Study 1 (Study 1: t_(57)_ = 3.40, p = 0.001). Although the mean proportion of LL choices in the Precommitment task was greater than in the Choice task in Study 2, the difference was not significant (t_(19)_ = 1.00, p = 0.328), likely due to the reduced sample size compared with Study 1. The task-related pattern of choices was consistent across delays (i.e., the task × delay interaction was not significant, Study 1: F_(6,342)_ = 1.16, p = 0.330; Study 2: F_(6,114)_ = 1.10, p = 0.369).

The improvement in self-control observed in the Precommitment task varied across subjects, such that more impulsive individuals were more likely to benefit from precommitment. We defined impulsivity, here, as breakdown of willpower; impulsivity was therefore estimated as the proportion of SS choices in the Willpower task. Improved self-control in the Precommitment task (defined as the difference between the proportion of LL choices in the Precommitment task and the average proportion of LL choices across the other tasks) was positively correlated with impulsivity (Study 1: r = 0.62, p < 0.001; Study 2: r = 0.50, p = 0.020).

### Willpower Engages DLPFC, IFG, and PPC

**Figure 3:**
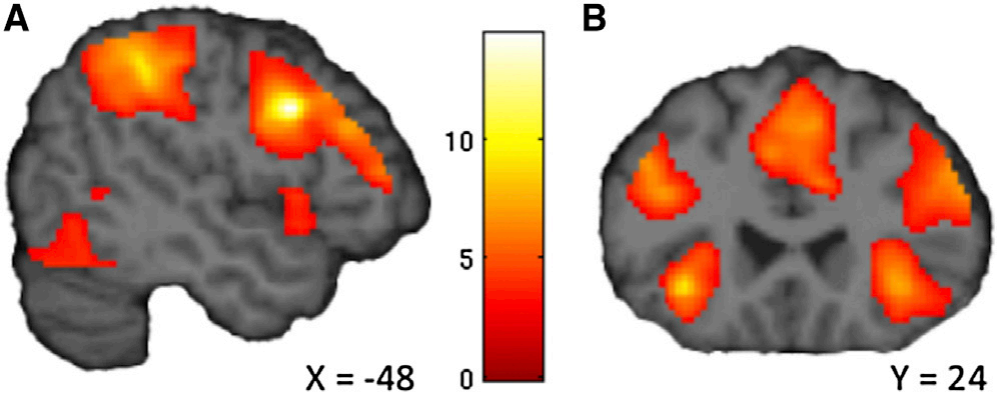
Willpower-Related Activations (A) Bilateral DLPFC, bilateral PPC, and (B) bilateral IFG were more activated when the temptation to choose the SS during the delay had to be suppressed. Images are displayed at a threshold of p < 0.005 uncorrected with an extent of >10 voxels. See also Table S2.

To identify brain regions involved in the effortful inhibition of impulses, we examined neural activity during the delay period. Such regions should be more engaged during delays in which participants must actively resist the temptation to choose SS, relative to delays in which the tempting SS option is absent. We compared blood oxygen level-dependent (BOLD) activity during the delay period in the Willpower task, in which subjects must continually resist the temptation to select the available SS, with activity during the delay period in the Choice task, in which the SS option was not available. Because we were interested in effective implementations of self-control, we restricted this analysis to trials with LL outcomes only, thus controlling for reward anticipation and delivery across conditions. We expected to find brain regions that have been previously associated with inhibition of prepotent responses, executive function, and self-control (McClure et al., 2004, 2007; Hare et al., 2009; Figner et al., 2010; Kober et al., 2010; Cohen et al., 2012; Essex et al., 2012; Luo et al., 2012). Confirming our hypothesis, this analysis revealed significant activations in bilateral DLPFC (peak −50, 10, 32; t_(19)_ = 14.39, p < 0.001, whole-brain family-wise error [FWE] corrected), bilateral IFG (peak −44, 42, 10; t_(19)_ = 6.44, p < 0.001, whole-brain FWE corrected), and bilateral PPC (peak −32, −52, 44; t_(19)_ = 8.80, p < 0.001, whole-brain FWE corrected) when subjects actively resisted temptations (Figure 3; Table S2). Additional willpower-related activations were observed in the cerebellum, ventral striatum, insula, posterior cingulate cortex, and parahippocampal gyrus (p < 0.05 whole-brain FWE corrected; Table S2).

### Precommitment Engages LFPC

**Figure 4:**
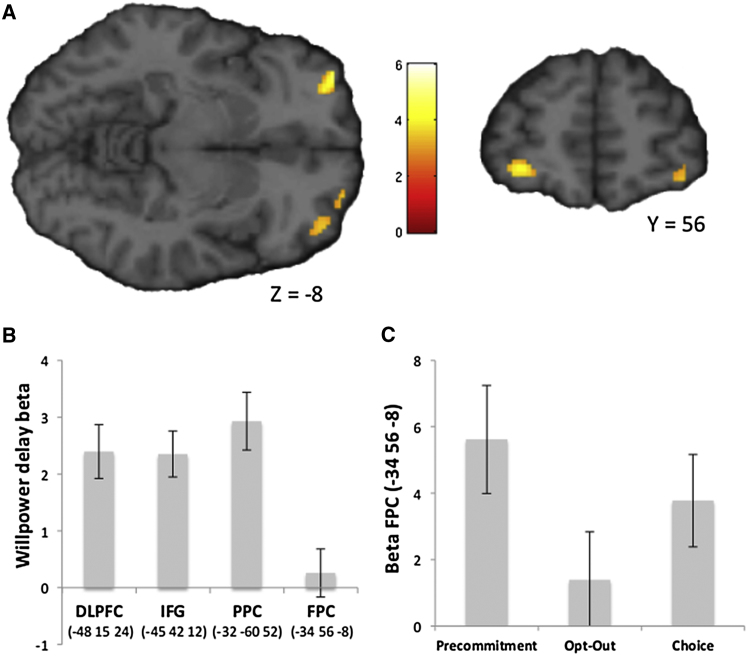
Precommitment-Related Activations (A) LFPC was activated when participants made binding choices for LL rewards, relative to nonbinding choices for LL rewards. (B) LFPC was not significantly activated when subjects actively resisted temptations during the delay period of the Willpower task (relative to the delay period of the Choice task). (C) In the decision tasks, LFPC activation was sensitive to the opportunity to make binding choices for delayed rewards. Images are displayed at a threshold of p < 0.005 uncorrected with an extent of >10 voxels. Data are represented as mean ± SEM. See also Figure S1 and Table S3.

To investigate the neural correlates of precommitment, we compared BOLD activity at decision onset during binding LL decisions in the Precommitment task with activity at decision onset during nonbinding (but otherwise identical) LL decisions in the Opt-Out task. Again, we restricted this analysis to choices with LL outcomes only, to control for reward anticipation across conditions. In line with our predictions, this analysis revealed activity in left and right LFPC (peak −34, 58, −8; t_(19)_ = 4.74, p = 0.014, small-volume FWE corrected; Figure 4A and Table S3).

We performed additional analyses to test the selectivity of LFPC activation to trials with opportunities to precommit. As in our previous analyses, we focused on trials in which subjects chose LL to control for reward anticipation across conditions. First, we investigated whether the LFPC showed sustained activation when subjects actively resisted temptations by extracting the Willpower contrast estimate from our region of interest (ROI) in LFPC (−34, 56, −8; Boorman et al., 2009). LFPC activation was not significantly different from zero when subjects actively resisted temptations (beta = 0.2653, SE = 0.4249, t_(19)_ = 0.64, p = 0.5294; Figure 4B). Directly contrasting BOLD responses from Precommitment trials in which subjects chose to precommit, against BOLD responses from Willpower trials in which subjects actively resisted temptations, revealed a significant cluster in right LFPC (40, 56, −12; t_(19)_ = 4.78, p = 0.039, whole-brain FWE corrected) and a trend-level significant cluster in left LFPC (−26, 52, −12; t_(19)_ = 5.11, p = 0.059, whole-brain FWE corrected).

Next, we examined the LFPC’s involvement in the three tasks involving explicit decisions (Precommitment, Choice, and Opt-Out). We extracted parameter estimates from our ROI in LFPC based on a previous study (−34, 56, −8; Boorman et al., 2009) for LL decisions in the three decision tasks and conducted a repeated-measures ANOVA to compare LFPC activation across tasks (Figure 4C). This analysis demonstrated a significant main effect of task on LFPC activity (F_(3,17)_ = 5.573, p = 0.008). Pairwise post hoc comparisons revealed that LFPC activation was significantly greater during precommitment choices than during LL choices in the Opt-Out task (t_(19)_ = 3.83, p = 0.003, Bonferroni corrected). The LFPC mean parameter estimate for precommitment choices was also greater than that for LL choices in the Choice task, but the difference did not survive correction for multiple comparisons, mirroring our behavioral self-control findings (compare Figure 4C with Figure 2C). We note that the Choice task, like the Precommitment task, also involves the opportunity to make a binding choice for LL; our results therefore support the notion that the LFPC is sensitive to the opportunity to make binding choices for large, but delayed, rewards.

For comparison, we also investigated whether regions involved in willpower (DLPFC, IFG, and PPC) were sensitive to opportunities to precommit. We extracted parameter estimates from these regions (using ROI coordinates from previous studies; Table S8) during LL choices in the three decision tasks and subjected them to a repeated-measures ANOVA. None of these regions were sensitive to opportunities to precommit (Figure S1); the effect of task was not significant for DLPFC (F_(3,17)_ = 1.676, p = 0.215), IFG (F_(3,17)_ = 1.209, p = 0.322), or PPC (F_(3,17)_ = 0.924, p = 0.415). Thus, DLPFC, IFG, and PPC showed activation patterns consistent with their role in self-control more generally but were not sensitive to opportunities to precommit.

Finally, we subjected the parameter estimates from LFPC, DLPFC, IFG, and PPC for the three decision tasks to a repeated-measures ANOVA with region and task as within-subjects factors. Parameter estimates were z transformed to control for differences in mean parameter estimates across regions. This analysis revealed a significant interaction between region and task (F_(6,114)_ = 3.989, p = 0.001), confirming our above observations that the LFPC was differentially activated across decision tasks, but the regions engaged during willpower (DLPFC, IFG, and PPC) were not.

### Functional Connectivity with LFPC during Precommitment

**Figure 5:**
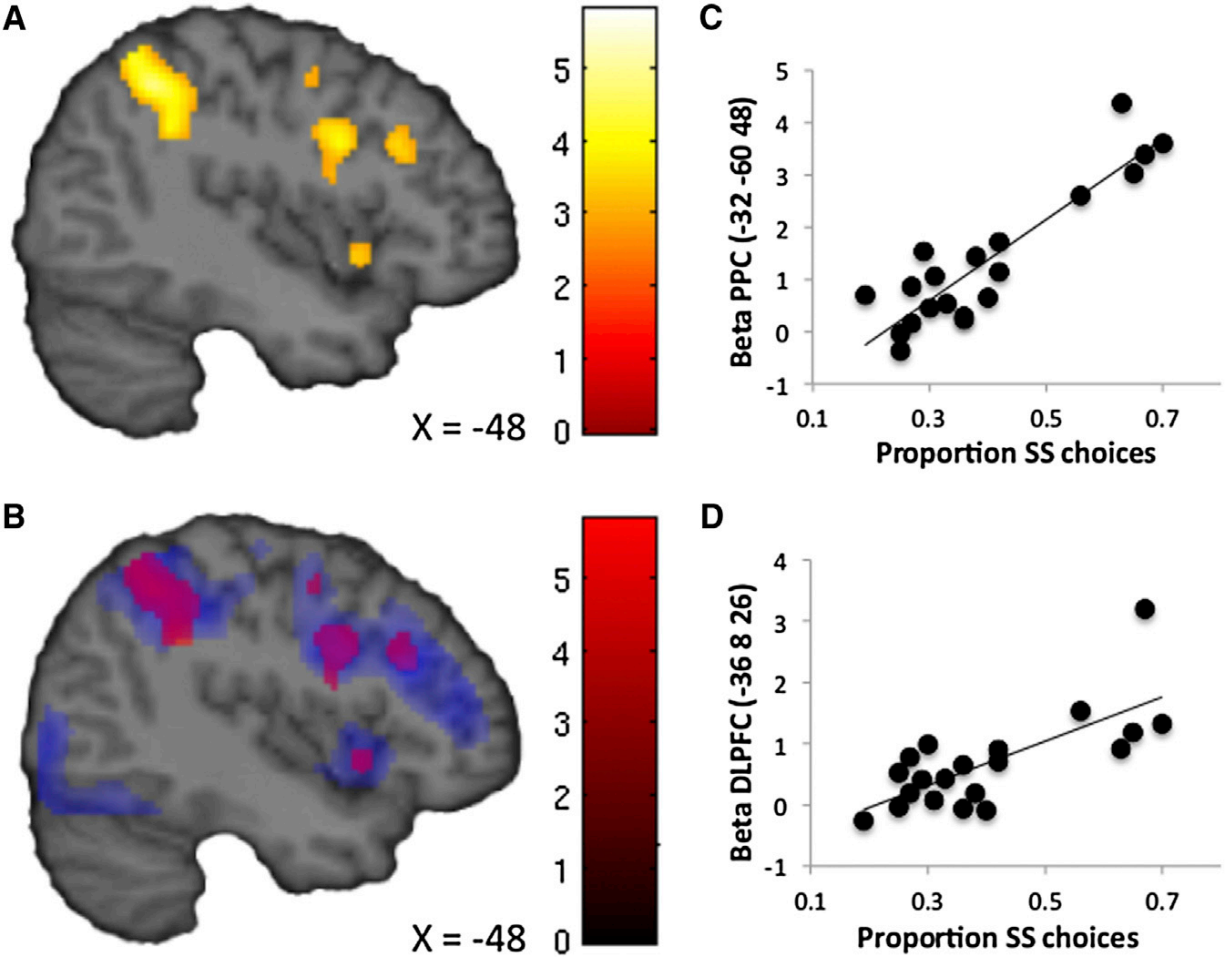
Positive Functional Connectivity with LFPC during Precommitment (A) The PPI analysis showed that activity in DLPFC and PPC correlated positively with the LFPC seed during precommitment. (B) Conjunction analysis revealed that regions showing positive functional connectivity with LFPC during precommitment (red) overlapped with regions activated during willpower (blue). Images are displayed at a threshold of p < 0.005 uncorrected with an extent of >10 voxels. (C) Individual differences in impulsivity were positively correlated with the strength of connectivity between LFPC and PPC (r = 0.90, p < 0.001). (D) Individual differences in impulsivity were positively correlated with the strength of connectivity between LFPC and DLPFC (r = 0.72, p < 0.001). The correlation remains significant when excluding the individual in the upper-right quadrant (r = 0.663, p = 0.002). See also Tables S4 and S6.

We next investigated the possibility that LFPC implements decisions to precommit by controlling activity in the DLPFC, in line with theories positing that the LFPC sits at the top of a cognitive control hierarchy from which it orchestrates different courses of actions represented in DLPFC (Tsujimoto et al., 2011; Koechlin and Hyafil, 2007; Burgess et al., 2007). This idea is particularly intriguing because of the DLPFC’s prominent role in actively implementing self-control (Hare et al., 2009). To test this hypothesis, we conducted a psychophysiological interaction (PPI) analysis with the seed in the LFPC cluster associated with precommitment to identify regions showing increased functional connectivity with LFPC at decision onset. The PPI analysis identified precommitment-related increases in positive functional connectivity between the LFPC and several regions identified in our willpower analysis, including DLPFC (t_(19)_ = 4.23, p = 0.016, small-volume FWE corrected), PPC (t_(19)_ = 5.78, p < 0.001, whole-brain FWE corrected), cerebellum (t_(19)_ = 5.44, p = 0.006, whole-brain FWE corrected), and middle frontal gyrus (t_(19)_ = 5.10, p = 0.011, whole-brain FWE corrected; Figure 5A and Table S4). A conjunction analysis confirmed that these were indeed the same regions as those engaged during willpower (Figure 5B). Thus, during precommitment decisions, the LFPC increased functional coupling with regions also involved in willpower.

### Reward Circuitry Encodes the Expected Value of Precommitment

Our behavioral analysis revealed that more impulsive individuals were more likely to benefit from precommitment; in other words, the expected value of precommitment differed across individuals. This suggests that brain regions associated with value computation should be engaged differentially during precommitment as a function of impulsivity. We tested this hypothesis by searching for precommitment-related brain regions that tracked individual differences in impulsivity (defined by proportion of SS choices in the Willpower task). To do this, we regressed individual differences in impulsivity onto the precommitment contrast (binding LL choices in the Precommitment task relative to nonbinding LL choices in the Opt-Out task). Note that the regressor used in this analysis was computed from choices on different trials than those used in the fMRI contrast. This analysis revealed significant clusters in the ventral striatum (t_(19)_ = 7.62, p < 0.001, whole-brain FWE corrected) and vmPFC (t_(19)_ = 4.91, p = 0.003, whole-brain FWE corrected; Table S5), regions previously associated with reward anticipation (Haber and Knutson, 2010).

### Impulsivity Moderates LFPC Connectivity during Precommitment

If the LFPC implements precommitment decisions as a function of expected value, we might expect functional connectivity between LFPC and willpower regions to differ as a function of individual differences in the expected value of precommitment. Since individuals varied in the extent to which they could benefit from precommitment, we were able to examine whether these individual differences predicted functional connectivity between LFPC and willpower regions. We conducted an ROI analysis by extracting individual mean parameter estimates from clusters in PPC and DLPFC identified independently in the previous PPI analysis (10 mm spheres surrounding the coordinates in Table S6) and regressed these values against individual differences in impulsivity (as defined in our previous analysis of expected value). This analysis revealed that more impulsive individuals indeed showed stronger functional connectivity during precommitment between the LFPC and PPC (r = 0.90, p < 0.001; Figure 5C) and between the LFPC and DLPFC (left: r = 0.72, p < 0.001; right: r = 0.52, p = 0.019; Figure 5D). For completeness, we also conducted a whole-brain analysis by regressing individual differences in impulsivity onto the PPI contrast. This analysis again revealed stronger positive LFPC coupling with PPC and DLPFC in more impulsive individuals, as well as IFG, MFG, and cerebellum (all p < 0.05, whole-brain FWE corrected; Table S6).

**Figure 6:**
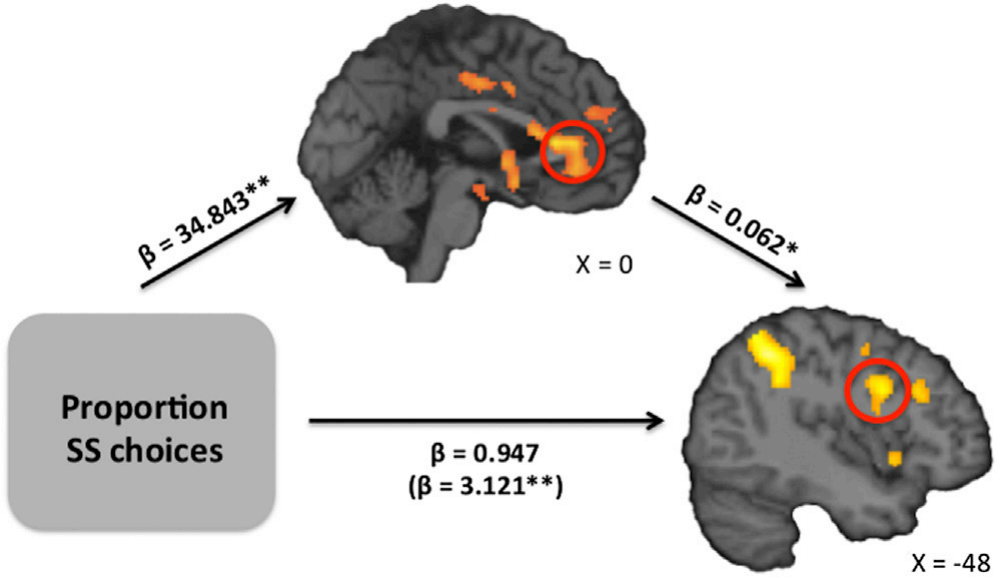
Mediation Analysis: Impulsivity, vmPFC Activation, and LFPC Connectivity vmPFC activation during precommitment (relative to LL choices in the Opt-Out task) mediated the relationship between impulsivity (defined as the proportion of SS choices in the Willpower task) and functional connectivity between LFPC and DLPFC during precommitment (relative to LL choices in the Opt-Out task). ^∗^p < 0.05, ^∗∗^p < 0.01. See also Tables S4, S5, and S6.

So far the data have shown that individual differences in impulsivity are positively correlated both with activation in reward circuitry during precommitment and with connectivity between LFPC and willpower regions during precommitment. These findings suggest that the LFPC implements precommitment decisions by driving activation in willpower regions and does so as a function of the expected value of precommitment. To further test this hypothesis, we examined whether activation in the vmPFC during precommitment (Table S5) mediated the relationship between impulsivity and LFPC-DLPFC connectivity during precommitment (Figure 5D). To avoid nonindependence concerns, we extracted parameter estimates from a region of vmPFC identified from a previous study (Kable and Glimcher, 2007). Using hierarchical regression (Baron and Kenny, 1986), we first demonstrated that vmPFC activation during precommitment significantly correlated with LFPC-DLPFC connectivity during precommitment (t_(19)_ = 2.668, p = 0.016). A second regression showed that impulsivity (proportion of SS choices during the Willpower task) significantly correlated with vmPFC activation during precommitment (t_(19)_ = 4.583, p = 0.002). Impulsivity also correlated with LFPC-DLPFC connectivity during precommitment (t_(19)_ = 3.576, p = 0.002). Importantly, adding vmPFC activation as a second predictor of LFPC-DLPFC connectivity removed the effect of impulsivity (p = 0.405), and the indirect effect of vmPFC activation on LFPC-DLPFC connectivity was significant (Z = 2.42, p = 0.016), consistent with a mediating role (Figure 6).

Thus, our findings suggest a functional model whereby the vmPFC evaluates the expected value of precommitment and relays this information to LFPC, which then implements those decisions via the DLPFC and PPC. Such a model would also imply an increase in functional connectivity between vmPFC and LFPC during precommitment, again as a function of the expected value of precommitment. This was indeed the case; our PPI model with the seed in LFPC showed an increase in LFPC-vmPFC connectivity during precommitment as a function of impulsivity (peak −8, 40, 6; t_(19)_ = 6.33, p = 0.01, small-volume FWE corrected; Table S6).

## Discussion

We provide behavioral evidence demonstrating that precommitment is an effective strategy for promoting self-control. In two independent studies, participants were more likely to obtain superior but delayed rewards when they had the opportunity to make a binding choice for the delayed option in advance, relative to when they simply had to wait for the delayed reward in the presence of a tempting inferior option. Notably, our experimental setting provided a tightly controlled comparison of the effectiveness of different self-control strategies: different task conditions were economically equivalent in terms of rewards, delays, and trial durations. Nevertheless, participants were less likely to receive large delayed rewards when they had to actively resist smaller-sooner rewards (Mischel et al., 1989), compared to when they could precommit to choosing the larger reward before being exposed to temptation (Ainslie, 1974).

Consistent with previous research (McClure et al., 2004, 2007; Hare et al., 2009; Figner et al., 2010; Kober et al., 2010; Cohen et al., 2012; Essex et al., 2012; Luo et al., 2012), we found that effortful inhibition of the impulse to choose a tempting but inferior reward was associated with strong activation in the DLPFC, IFG, and PPC during the waiting period. Precommitment was associated with activation in the LFPC. The LFPC was more active during precommitment than during willpower and was more active when subjects had the opportunity to make binding (relative to nonbinding) choices for LL rewards. These activation patterns suggest that the LFPC is sensitive to the presence of opportunities to precommit and may play a role in deciding whether to precommit.

The LFPC has been previously associated with metacognition, counterfactual thinking, and prospective valuation (Daw et al., 2006; De Martino et al., 2013; Gilbert et al., 2006; Burgess et al., 2007; Koechlin and Hyafil, 2007; Boorman et al., 2009, 2011; Charron and Koechlin, 2010; Rushworth et al., 2011; Tsujimoto et al., 2011). These cognitive processes are all expected to play a role in precommitment, which may involve recognizing, based on past experience, that future self-control failures are likely if temptations are present. Previous studies of the LFPC suggest that this region specifically plays a role in comparing alternative courses of action with potentially different expected values (Daw et al., 2006; Boorman et al., 2009, 2011; Rushworth et al., 2011), a process that may rely on prospective (“look-ahead”) working memory capacity (Koechlin and Hyafil, 2007; Charron and Koechlin, 2010). Our findings provide further support for this hypothesis in the context of self-controlled decision making.

A functional connectivity analysis demonstrated that during precommitment decisions, the LFPC showed increased coupling with the DLPFC and PPC. These regions have consistently been implicated in willpower, both in the current study and many others (McClure et al., 2004, 2007; Hare et al., 2009; Figner et al., 2010; Kober et al., 2010; Cohen et al., 2012; Essex et al., 2012; Luo et al., 2012). The LFPC may therefore access information about the strength of willpower processes from the DLPFC and PPC when assessing the potential benefits of precommitment. Previous fMRI studies of self-control suggest that the DLPFC promotes self-control by enhancing the weight of long-term goals in the neural computation of outcome values (Hare et al., 2009). The LFPC may therefore integrate information about long-term goals provided by the DLPFC when assessing the potential benefits of precommitment. Meanwhile, the PPC may be involved in the implementation of precommitment decisions, acting as an interface between value computations and motor outputs. Two previous studies have reported coactivation of the LFPC and the PPC during exploratory decision making (Daw et al., 2006; Boorman et al., 2009); in these studies, activation in the PPC predicted switches in behavioral strategies. Taken together, and consistent with cognitive hierarchy models of action control (Burgess et al., 2007; Koechlin and Hyafil, 2007; Tsujimoto et al., 2011), these results suggest that the LFPC orchestrates precommitment by translating precommitment values into actions via the PPC.

The benefits of precommitment were stronger for participants with weak willpower, suggesting that precommitment may be a viable alternative self-control strategy when willpower is constitutively weak or situationally depleted. Neuroimaging data showed that participants with weaker willpower displayed stronger activation in the ventral striatum and vmPFC during binding choices for larger delayed rewards, relative to nonbinding choices for larger delayed rewards. These regions have been consistently implicated in the computation of expected value (Haber and Knutson, 2010), suggesting that those who stand to benefit more from precommitment encode those benefits more strongly in the brain’s reward circuitry. This result supports the idea that individuals possess a degree of self-knowledge about their own self-control abilities—information they may use when deciding whether to precommit—and fits with previous studies implicating the LFPC in metacognition (Fleming et al., 2010; De Martino et al., 2013) and the representation of anticipatory utility during intertemporal choice (Jimura et al., 2013).

Notably, impulsive participants who stood to benefit more from precommitment—those who were more likely to succumb to temptation when attempting to exert willpower—showed stronger positive connectivity between LFPC and willpower regions during precommitment, relative to their cooler-headed peers. Moreover, activation in the vmPFC during precommitment mediated the relationship between impulsivity and LFPC-DLPFC connectivity. These findings suggest that LFPC adaptively implements precommitment decisions as a function of their expected value, consistent with its hypothesized role in calculating the value of alternative courses of action (Boorman et al., 2009; Rushworth et al., 2011).

Theoretical models predict that precommitment arises as a function of learning about one’s own self-control abilities (Kurth-Nelson and Redish, 2010, 2012; Ali, 2011). In the current study, we were able to show that between-subject differences in self-control abilities moderated precommitment-related neural activity. Future work might examine the within-subject dynamics of learning about one’s own self-control abilities and how such learning relates to precommitment. For example, one might dynamically manipulate the difficulty of resisting temptations (thus making precommitment more valuable at some times than others) and examine how activation in LFPC and its connectivity with willpower regions tracks with the expected value of precommitment on a trial-to-trial basis. The LFPC may be involved in such learning processes, given its role in self-awareness and metacognition (Fleming et al., 2010; De Martino et al., 2013).

Although the anterior prefrontal cortex (BA 10) is cytoarchitechtonically homogeneous, it may be functionally heterogeneous (Gilbert et al., 2006; Liu et al., 2013); for instance, studies of metacognition (Fleming et al., 2010; De Martino et al., 2013) have reported activations in anterior prefrontal cortex that are situated dorsal and medial to those reported in studies of counterfactual value processing (Boorman et al., 2009, 2011). A recent study of connectivity patterns within FPC found that the lateral FPC (FPCl) showed strongest connectivity to DLPFC, while the orbital FPC (FPCo) showed strongest connectivity to the OFC and subgenual ACC (Liu et al., 2013). Notably, the region we found to be associated with precommitment is located precisely in the transition zone between FPCl and FPCo. This region is therefore ideally situated to arbitrate between regions involved in calculating expected value (OFC, subgenual ACC) and regions involved in implementing self-control (DLPFC). Fitting with this notion, we observed that LFPC was functionally connected to DLPFC during precommitment and that the strength of this connectivity was moderated by activation in the vmPFC.

Precommitment decisions in the real world often involve longer delays (in the order of weeks to months), in contrast with the shorter delays used in the current study. Future studies might examine whether the precommitment to large rewards with much longer delays engage similar neural processes as those described in the current study. Given the role of the LFPC in forward planning (Daw et al., 2006; Burgess et al., 2007; Koechlin and Hyafil, 2007; Boorman et al., 2009, 2011; Rushworth et al., 2011; Tsujimoto et al., 2011), we might expect to see even stronger effects in LFPC with longer delays than in our current design, in which the shorter delays placed relatively low demands on prospective cognition.

Self-control problems characterize a number of counterproductive behaviors, including substance abuse, overeating, overspending, and procrastination. It remains unclear whether these problems are the result of poor willpower, impaired ability to precommit, or some mixture of both. Our method for measuring willpower and precommitment in the same individuals offers promising new avenues for understanding the mechanisms underlying self-control failures in the context of drug abstinence, dieting, saving, and studying. Our behavioral paradigm could be adapted to study self-control deficits in specific groups (e.g., replacing erotic pictures with desirable foods to study self-control in dieters). Knowing whether self-control failures stem from impaired willpower versus precommitment in various clinical populations could inform the development of targeted behavioral or pharmacological interventions aimed at improving function in the impaired faculty.

Finally, our finding that the ability to precommit facilitates the pursuit of long-term goals has potential practical implications. If organizations wish to promote future-minded decisions, they could achieve this by providing opportunities to commit to delayed rewards in advance. One famous example already in place is the “Save More Tomorrow” scheme, which enables employees to commit in advance allocations of future raises toward retirement savings (Thaler and Sunstein, 2008). Entrepreneurs have also realized that people value commitment opportunities and are developing digital applications like SelfControl (http://selfcontrolapp.com), which allows users to specify in advance which websites they wish to prohibit their future selves from browsing. Humans may be woefully vulnerable to self-control failures, but thankfully, we are sometimes sufficiently far-sighted to circumvent our inevitable shortcomings.

## Experimental Procedures

### Participants

Healthy right-handed heterosexual males from Cambridge (n = 78, Study 1) and Amsterdam (n = 28, Study 2), aged 18–35, gave informed consent and participated in the study that was approved by the local departmental ethics committee at the University of Cambridge (Study 1) and the University of Amsterdam (Study 2). Participants were recruited through the general public as well as the Universities of Cambridge and Amsterdam. Exclusion criteria included current or past drug use, psychiatric or neurological disorders, MRI contraindications, and red-green colorblindness. In Study 1 (Cambridge), we excluded participants whose ratings of the stimulus set did not provide sufficient variation to construct the required number of SS and LL stimuli (see below for details); 58 subjects were available for analysis. In Study 2 (Amsterdam), potential subjects rated the stimulus set online and only those whose ratings allowed us to construct the required number of SS and LL stimuli were invited for scanning. One subject was excluded due to a large temporal lobe cyst revealed by the structural image. Two subjects were excluded for revealing a recent use of recreational drugs. Two subjects were excluded due to a programming error that resulted in a loss of task data. One subject was excluded due to a back-wrapping artifact in the fMRI images that prevented successful normalization. Finally, two subjects were excluded for excessive movement in the scanner (>5 mm; all other subjects had movement <3 mm). Twenty subjects were therefore available for the fMRI analysis.

### Self-Control Task

In both experiments, participants made choices between smaller-sooner rewards (SS) and larger-later rewards (LL) in four experimental task conditions (Figure 1). Each condition had 42 trials, for a total of 168 trials. The trials were presented across six runs, each consisting of blocks of seven trials of all four experimental conditions, presented in random order within a run. Participants were trained on all four task conditions before commencing the experiment. Each condition was assigned a different color, which we used to alert subjects to the upcoming condition at the start of each block (e.g., “green task,” “red task,” “yellow task,” and “blue task”). The assignment of color to task condition was counterbalanced across subjects.

In all task conditions, participants faced choices between SS and LL rewards. If the SS reward was chosen, an SS image was displayed immediately for 2,500 ms. If the LL reward was chosen, an LL image was displayed for 2,500 ms after a variable delay, which could be short (∼4,000 ms), medium (∼7,000 ms), or long (∼10,000 ms). We used relatively short, experienced delays in order to be able to capture neural activation as subjects endured the entirety of the delay period (Prévost et al., 2010). Each condition consisted of 12 short, 18 medium, and 12 long trials. We included a higher number of medium trials because pilot testing indicated that choices for LL were most variable at medium delays. The length of the LL delay (short, medium, or long) was indicated at the time of choice. Importantly, we further adjusted the length of the intertrial interval (ITI) to fix the total length of each trial at 19,000 ms, regardless of whether the SS or the LL was chosen. Participants therefore could not finish the task more quickly by choosing SS reward and were instructed explicitly about this. Thus, to maximize reward in this paradigm, participants should always choose LL.

All task conditions consisted of an initial *decision phase* (4,000 ms), a *delay phase* (0–10,000 ms), a *reward delivery phase* (2,500 ms), and an *ITI* (at least 1,000 ms; mean depended on subjects’ decisions). During the decision phase, participants indicated their choice. If participants chose the SS, they immediately entered the reward delivery phase (i.e., delay = 0), followed by the ITI. If participants chose to wait for the LL, they entered the delay phase. At the end of the delay, participants could “collect” the reward by selecting the LL, at which point they entered the reward delivery phase, followed by the ITI. Delay and ITI lengths were variable (jittered) so that we could separate BOLD responses associated with the decision phase, the delay phase, and the reward phase.

For half the trials, the SS option was displayed on the left of the screen, and the LL option was displayed on the right of the screen, with these positions reversed for the other half of trials. Participants indicated their choices with left- and right-button presses via keyboard (Study 1) or button box (Study 2).

### Experimental Task Conditions

In the Willpower task (Figure 1A), we measured the effortful inhibition of impulses to choose the SS. Participants did not make an explicit choice during the initial phase but pressed a third key to enter the delay phase. Upon entering the delay phase, the SS reward became available for selection, remaining so for the duration of the delay. The LL reward was not available for selection until the end of the delay phase. Participants could terminate the delay phase at any time by selecting the SS, at which point they entered the reward delivery phase, followed by the ITI. In order to select the LL reward, participants had to resist the temptation to choose the available SS for the duration of the delay until the LL reward became available.

In the Choice task (Figure 1B), participants initially made a simple choice between LL and SS during the decision phase. If SS was chosen, participants entered the reward delivery phase, followed by the ITI. If LL was chosen, participants entered the delay phase, followed by the reward delivery phase and the ITI. Critically, the SS was not available during the delay phase of the Choice task. Thus, contrasting neural activity during the delay phase of the Willpower task (in which the SS was available) with neural activity during the delay phase of the Choice task should yield brain regions associated with the effortful inhibition of impulses to choose the SS, controlling for LL reward anticipation (which is matched across conditions).

In the Precommitment task (Figure 1C), which was inspired by the animal literature (Rachlin and Green, 1972; Ainslie, 1974), during the decision phase participants chose whether or not to make a binding choice for the LL (“commit”). If participants chose to commit, they entered a delay phase during which the SS was not available, followed by the reward delivery phase and the ITI. If participants chose not to commit, they entered a delay phase during which the SS was available for the duration of the delay, as in the Willpower task. Thus, by choosing to commit, participants restricted their access to the SS option during the delay period.

In the Opt-Out task (Figure 1D), participants made an initial choice between LL and SS during the decision phase. If SS was chosen, participants entered the reward delivery phase, followed by the ITI. If LL was chosen, participants entered the delay phase during which the SS was available for the duration of the delay, as in the Willpower task. Thus, choosing LL in this task was not a binding choice, as participants could still “opt out” of their initial choice by selecting SS at any point during the delay. Contrasting neural activity during binding commitment choices in the Precommitment task with nonbinding LL choices in the Opt-Out task should yield brain regions associated with precommitment, controlling for LL reward anticipation (which is matched across conditions).

### Stimuli

Because our self-control task used experiential delays, for rewards we used primary reinforcers that were consumable at the time of delivery, as is common practice in the animal literature. We chose to use erotic images, based on a previous study that examined temporal discounting with experiential delays in humans (Prévost et al., 2010). Erotic images have advantages over alternative primary reinforcers, such as juice or food rewards (e.g., McClure et al., 2004), in an fMRI setting. The consumption of edible rewards can create fMRI movement artifacts; there may be individual variability in preferences for the rewards, creating between-subject variability in hedonic value; and subjects can become satiated on the reward. Using erotic pictures enabled us to sidestep these issues. We were able to construct individualized stimulus sets for each subject, to match the subjective value of SS and LL rewards, thus minimizing between-subject variability in the hedonic value of the stimuli. Furthermore, we minimized the problem of satiation by never showing the same image more than once.

Prior to completing the self-control task, participants provided pleasure ratings on a Likert scale of 0–10 for a set of 400 images of women in lingerie and swimwear (300 × 380 pixels, 24 bit color depth). We explicitly instructed participants that a rating of 0 indicated that the image was not enjoyable, a rating of 1 indicated neutral feelings toward the image, and ratings of 2–10 indicated that the image was enjoyable (with 10 being most enjoyable). For each participant, we discarded all images rated 0 or 1 and computed the median rating for the remaining images. We then designated images rated above the median as LL rewards and those rated below the median as SS rewards (Figure S2). Each participant thus received a personalized set of stimuli, with LL rewards as their more highly rated images and SS rewards as less highly but still positively rated images. Each stimulus set contained a sufficient number of SS and LL images such that no image would be presented more than once throughout the duration of the experiment (and subjects were explicitly informed of this).

We note that all images used are freely available on the Internet. However, subjects did not have free access to the images during testing, so they are likely to have valued them highly at the time of delivery. This claim is corroborated by subjects’ self-reports and neural activity. The ratings for LL images were significantly higher than for SS images (Exp. 1: t(57) = 44.276, p < 0.0001; Exp. 2: t(19) = 27.200, p < 0.0001; Table S1). A categorical comparison of BOLD responses to LL reward onsets versus SS reward onsets indicated that the LL rewards activated ventromedial PFC and ventral striatum more strongly than SS rewards (Table S7), consistent with previous studies (Knutson et al., 2008; Prévost et al., 2010).

### Image Acquisition and Analysis

fMRIs were collected with a Phillips Intera 3.0T at the university hospital of the University of Amsterdam using a standard six-channel SENSE head coil and a T2^∗^ sensitive gradient echo (EPI) sequence (96 × 96 matrix, repetition time [TR] 2,000 ms, echo time [TE] 30 ms, flip angle [FA] 80°, 34 slices, 2.3 mm × 2.3 mm voxel size, 3-mm-thick transverse slices). Stimuli were presented using Eprime 1.2 software (Psychology Tools). The behavioral responses were collected by an fMRI-compatible four-button response box (Lumitouch).

All image preprocessing and analysis was carried out in SPM8 (Wellcome Department of Imaging Neuroscience). Images were realigned to the first scan of the first session, spatially normalized via segmentation of the T1 structural image into gray matter, white matter, and CSF using ICBM tissue probability maps, and spatially smoothed with a Gaussian kernel (8 mm, full-width at half-maximum).

We regressed fMRI time series onto a general linear model (GLM) with separate regressors for decision onsets, delay periods, and reward onsets. We modeled BOLD responses at decision onset as stick functions, conditioned by task and choice (Willpower: SS or LL; Choice: SS or LL; Precommitment: Commit, No Commit and choose SS, No Commit and wait for LL; Opt-Out: SS, LL). For trials in which participants initially began to wait for LL but chose SS during the delay period, we also modeled BOLD responses at SS choice onset as stick functions. We modeled BOLD responses at delay onset as boxcars set to the duration of the delay, conditioned by task and choice where appropriate (Willpower, Choice, Precommitment-Commit, Precommitment-No Commit, and Opt-Out). Finally, we modeled BOLD responses at reward onset as stick functions, separated by reward type (SS versus LL). The full model contained 17 regressors, each convolved with the canonical hemodynamic response function, plus six motion regressors of no interest, multiplied across six runs.

For the PPI analysis, we created an LFPC seed regressor by computing individual average time series within a 4 mm sphere surrounding individual subject peaks within the functional mask of left LFPC shown in Figure 4A. The location of the peak voxels was based on the contrast of commitment decisions in the Precommitment task versus LL choices in the Opt-Out task. Variance associated with the six motion regressors was removed from the extracted time series. To construct a time series of neural activity in left LFPC, the seed time courses were deconvolved with the canonical hemodynamic response function. We then estimated a PPI model with the following regressors: (1) an interaction between the neural activity in LFPC and a vector coding for the main effect of decision type (1 for Precommitment, −1 for Opt-Out LL); (2) the main effect of decision type; and (3) the original BOLD eigenvariate (i.e., the average time series from the LFPC seed), as well as six motion parameters as regressors of no interest.

To further investigate the results of the PPI analysis, we conducted a conjunction analysis by finding the intersection of voxels that were significant in the willpower contrast at p < 0.05 whole-brain cluster-level corrected and that also showed significant precommitment-related functional connectivity with LFPC at p < 0.001 uncorrected with an extent threshold of 10 voxels.

We tested for statistical significance using small-volume correction (p < 0.05, family-wise error corrected at the cluster level) in a priori regions of interest (ROIs) identified from the literature in DLPFC, IFG, PPC, and LFPC (Table S8). ROI masks were constructed as bilateral 10 mm spheres centered on peak coordinates from previous studies of value-based decision making (Supplemental Experimental Procedures). We also note results outside our regions of interest that survive whole-brain cluster-level corrections. Images are displayed at a threshold of p < 0.005, k > 10 to show the extent of activation in the significant clusters. Results are reported using the MNI coordinate system.

For the ROI analyses, we extracted contrast-specific parameter estimates for each ROI (identified from the literature, as above). To test for the effects of condition on responses in each ROI, we conducted repeated-measures ANOVA on the parameter estimates in SPSS v21. One subject was excluded from this analysis for having parameter estimates more than two SDs higher than the group mean. For the cross-region comparison ANOVA, we were not interested in differences in average parameter estimates across regions but rather in the within-region differences across tasks. We therefore first z transformed the parameter estimates for each region separately by subtracting each region × task parameter estimate from the mean parameter estimate for that region (collapsed across tasks) and dividing by the SD of the parameter estimates for that region across tasks.

For the mediation analysis, we used hierarchical linear regression as outlined in Baron and Kenny (1986). Indirect effects in the mediation model were estimated using the SPSS procedure described in Preacher and Hayes (2004). All parameter estimates used in the mediation analyses were extracted from coordinates derived from previous studies (Table S8) to avoid nonindependence issues. vmPFC parameter estimates were extracted from the Precommit > Opt-Out LL contrast. DLPFC parameter estimates were extracted from the PPI contrast (the interaction between the neural activity in the LFPC seed and a vector coding for the main effect of decision type [1 for Precommitment, −1 for Opt-Out LL]).

## Supplemental Information

Document S1. Figures S1 and S2, Tables S1–S8, and Supplemental Experimental Procedures
