# Supplementary material for: Restricting Temptations: Neural Mechanisms of Precommitment
Source: Neuron. 2013 Jul 24;79(2):391–401. doi: 10.1016/j.neuron.2013.05.028 (PMC3725418; doi:10.1016/j.neuron.2013.05.028)
Supplement: Document S1. Figures S1 and S2, Tables S1–S8, and Supplemental Experimental Procedures [file mmc1.pdf]

Neuron, Volume *79*

Supplemental Information

Restricting Temptations:

Neural Mechanisms of Precommitment

Molly J. Crockett, Barbara R. Braams, Luke Clark, Philippe N. Tobler, Trevor W. Robbins, and Tobias Kalenscher

Supplemental Data

Figure S1, related to Figure 4. Parameter estimates in (a) DLPFC, (b) IFG, and (c) PPC during LL choices across decision tasks

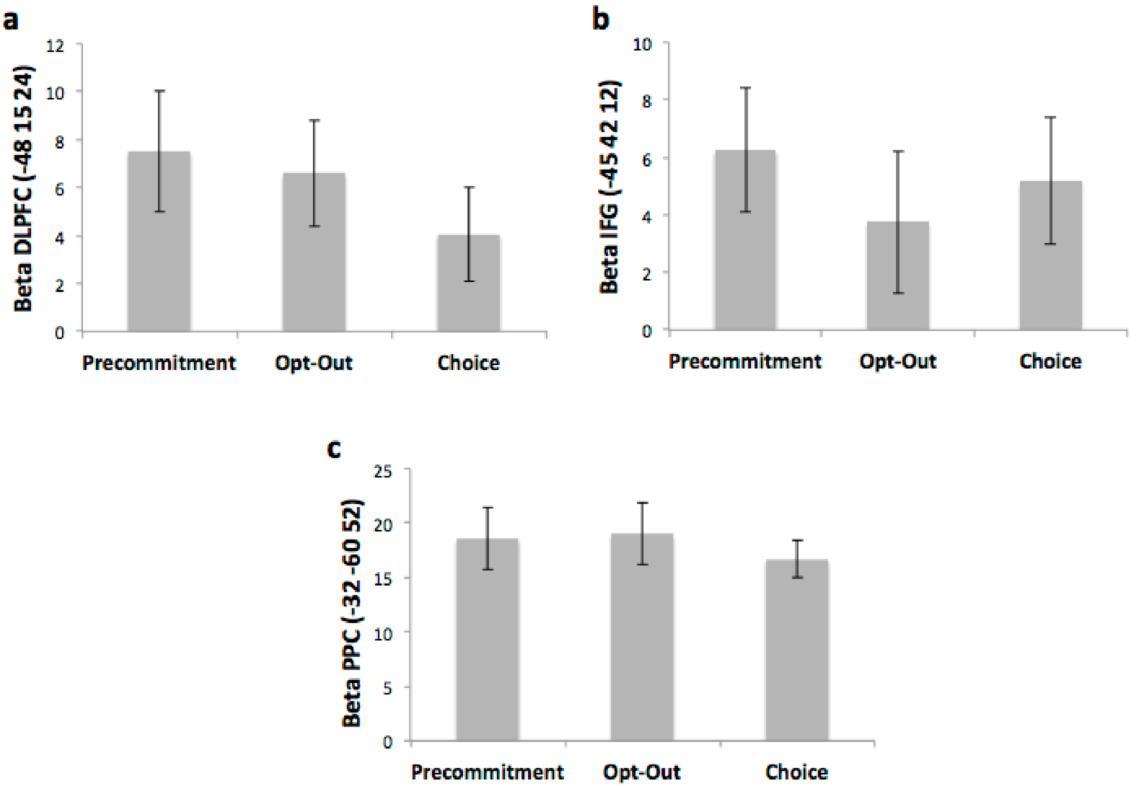

Table S1, related to Figure 1. Mean ratings for SS and LL stimuli.

	**Study 1**	**Study 2**
	**Mean ± S.E.**	**Mean ± S.E.**
**SS**	3.69 ± 0.18	3.67 ± 0.15
**LL**	7.07 ± 0.19	7.30 ± 0.22

Table S2, related to Figure 3. Regions associated with willpower

Region	L/R	X	Y	Z	k-extent*	p	T	*Z*	BA
DLPFC	L	-50	10	32	8560 (extends to IFG and DMPFC)	< 0.001***	14.39	6.79	9
	R	58	22	26		< 0.001***	7.62	5.10	9
IFG	L	-44	42	10		< 0.001***	6.44	4.74	46
	R	44	32	20		< 0.001***	7.67	5.12	46
IFG	L	-36	22	-6	800 (extends to insula)	< 0.001***	9.18	5.61	47
	R	32	28	-6	818	< 0.001***	8.03	5.24	47
PPC	L	-32	-52	44	16494 (extends to R)	< 0.001***	8.80	5.49	40
	R	36	-62	44		< 0.001***	7.92	5.21	7
Cerebellum	L	-26	-64	-22	16494	< 0.001***	10.28	5.91	-
VS	R	16	0	0	244	0.023***	5.58	4.24	-
PCC	R	6	-40	26	256	0.019***	5.50	4.20	23
PHG	L	-16	-32	-6	214	0.037***	5.32	4.11	30

DLPFC, dorsolateral prefrontal cortex; IFG, inferior frontal gyrus; PPC, posterior parietal cortex; VS, ventral striatum; PCC, posterior cingulate cortex; PHG, parahippocampal gyrus

*Thresholded at p<0.001 uncorrected
*** Whole brain corrected (cluster-level FWE)

Table S3, related to Figure 4A. Regions associated with precommitment

Region	L/R	X	Y	Z	k-extent*	p	T	Z	BA
LFPC	L	-34	58	-8	38	0.019**	4.74	3.80	10
	R	46	48	-14	10	0.001^{u}	3.92	3.32	10
DMPFC	R	12	26	50	117	0.001^{u}	6.01	4.44	8/9
	R	4	48	32	13	0.001^{u}	3.92	3.31	9
ACC	L	-2	12	26	17	0.001^{u}	4.52	3.68	33
MFG	R	40	16	48	100	0.001^{u}	4.36	3.58	8
SFG	R	24	38	46	20	0.001^{u}	4.28	3.54	8

LFPC, lateral frontopolar cortex; DMPFC, dorsomedial prefrontal cortex; ACC, anterior cingulate cortex; MFG, middle frontal gyrus; SFG, superior frontal gyrus

*Thresholded at p<0.001 uncorrected
** Small-volume corrected (cluster-level FWE)
^{u} peak-level uncorrected

Table S4, related to Figures 5A and 5B. Positive connectivity with LFPC during precommitment

Region	L/R	X	Y	Z	k-extent*	p-value	T	Z	BA
PPC[a]	L	-32	-60	48	1792	< 0.001***	5.78	4.34	7
DLPFC [a]	R	42	12	30	43	0.016**	4.23	3.50	9
	L	-36	8	26	92	0.046**	5.35	4.13	9
Cerebellum[a]	R	22	-86	-22	109	0.006***	5.44	4.18	-
MFG [a]	R	32	6	64	306	0.011***	5.10	4.00	6

PPC, posterior parietal cortex; DLPFC, dorsolateral prefrontal cortex; MFG, middle frontal gyrus

*Thresholded at p<0.001 uncorrected
*** Whole-brain corrected (cluster-level FWE)
** Small-volume corrected (cluster-level FWE)
[a] survives conjunction analysis of all voxels showing (1) significant activation during the delay period of the willpower task, relative to the choice task, $p < 0.05$, whole brain cluster-level corrected (Table S5) and (2) significant precommitment related functional connectivity with FPC at $p < 0.001$ uncorrected with an extent threshold of 10 voxels.

Table S5, related to Figure 6. Precommitment-related activity positively correlated with impulsivity

Region	L/R	X	Y	Z	k-extent*	p-value	T	Z	BA
VS	R	28	-6	-2	908	< 0.001***	7.62	5.03	-
VMPFC/ACC	R	2	30	10	314	0.003***	4.91	3.86	24
DMPFC	L	-12	56	24	179	0.039***	6.51	4.61	9

VS, ventral striatum; VMPFC, ventromedial prefrontal cortex; ACC, anterior cingulate cortex; DMPFC, dorsomedial prefrontal cortex

*Thresholded at p<0.001 uncorrected
*** Whole-brain corrected (cluster-level FWE)

Table S6, related to Figures 5C and 5D. Positive connectivity with LFPC during precommitment, moderated by impulsivity

Region	L/R	X	Y	Z	k-extent*	p-value	T	Z	BA
PPC[a]	R	32	-60	48	1584	< 0.001***	9.63	5.65	7
	L	-32	-58	46	2051	< 0.001***	7.56	5.01	7
DLPFC[a]	L	-46	2	24	619	< 0.001***	7.60	5.03	9
IFG[a]	R	40	34	16	219	0.022***	6.03	4.41	46
DMPFC[a]	L/R	0	20	48	679	0.008***	5.49	4.16	8
MFG[a]	L	-34	-2	54	342	0.003***	7.00	4.80	6
Cerebellum[a]	R	30	-48	-26	182	0.045***	6.05	4.41	-
vmPFC/ACC	L	-8	40	6	60	0.01**	6.33	4.54	32

PPC, posterior parietal cortex; DLPFC, dorsolateral prefrontal cortex; IFG, inferior frontal gyrus; MFG, middle frontal gyrus; VMPFC, ventromedial prefrontal cortex; ACC, anterior cingulate cortex; DMPFC, dorsomedial prefrontal cortex

*Thresholded at p<0.001 uncorrected
*** Whole-brain corrected (cluster-level FWE)
** Small-volume corrected (cluster-level FWE)
[a] survives conjunction analysis of all voxels showing (1) significant activation during the delay period of the willpower task, relative to the choice task, p < 0.05, whole brain cluster-level corrected (Table S5) and (2) significant precommitment related functional connectivity with FPC at p < 0.001 uncorrected with an extent threshold of 10 voxels.

Table S7. Regions more active for LL rewards than SS rewards

Region	L/R	X	Y	Z	k-extent*	p	T	Z	BA
VS	L	-2	10	-6	19451 (extends to VMPFC & insula)	< 0.001***	7.34	4.93	-
	R	2	10	-6	see VS	< 0.001***	6.76	4.71	-
VMPFC/ ACC	R	2	42	-2	see VS	< 0.001***	4.47	3.62	24
Insula	L	-30	24	2	see VS	< 0.001***	7.91	5.13	13
	R	24	24	6	205	0.025***	6.68	4.68	13
MFG	L	-30	32	24	405	0.001***	6.36	4.55	9
IFG	R	44	2	18	292	0.005***	5.75	4.28	9

VS, ventral striatum; VMPFC, ventromedial prefrontal cortex; ACC, anterior cingulate cortex; MFG, middle frontal gyrus; IFG, inferior frontal gyrus

*Thresholded at p<0.001 uncorrected
*** Whole-brain corrected (cluster-level FWE)

LFPC activation: Commit > Non-commit choices

We also investigated whether LFPC activation was greater during trials in which subjects chose to precommit, compared with trials on which subjects chose not to precommit. Ideally, we would be able to contrast precommitment trials against trials on which subjects chose not to commit, but still decided to wait for the LL reward – thus holding preferences for LL and reward anticipation constant across conditions. Unfortunately, we were unable to compute this contrast for a substantial number of subjects, due to an insufficient number of trials where subjects did not commit but nevertheless waited for the LL. We were able to contrast precommit trials against non-commitment trials (the majority of which resulted in subjects choosing the SS reward). This contrast revealed a trend-level activation in the LFPC (peak -32 48 -12, $t_{(19)} = 3.37$, $p = 0.069$, small volume FWE corrected). Although this finding is consistent with our main hypothesis, it is unclear whether this contrast reflects differences in LFPC activation as a function of precommitment, differences in preferences for LL vs. SS rewards, or differences in reward anticipation.

Supplemental Experimental Procedures

Table S8, related to Figures 3-6. Region of interest coordinates

ROI	X	Y	Z	Reference
DLPFC	±48	15	24	Hare et al., 2009
IFG	±45	42	12	Hare et al., 2009
PPC	±32	-60	52	McClure et al., 2004
LFPC	-34<br>36	56<br>54	-8<br>0	Boorman et al., 2009
VMPFC	0	44	0	Kable & Glimcher, 2007

Figure S2, related to Figure 1. Construction of stimulus set for an example subject

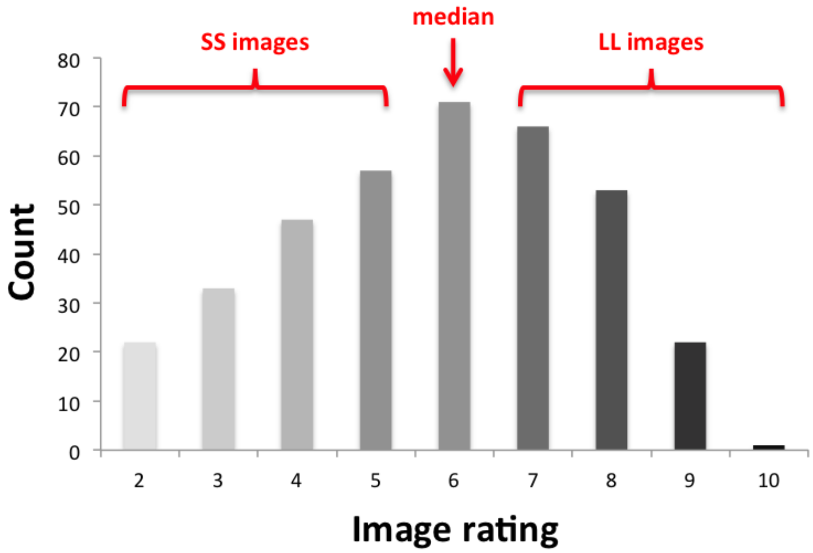

Participant 20 in Study 2 rated 372 out of 400 images as enjoyable (2 or higher on a scale of 0-10, with 0 being not enjoyable, 1 being neutral, and 2-10 being enjoyable). Figure S1 depicts a histogram of the ratings for these 372 images. The median rating was 6; we therefore discarded images with a rating of 6 from the stimulus set and designated images rated between 2-5 as SS rewards, and images rated between 7-10 as LL rewards.
